# Partial enzyme digestion facilitates regeneration of crushed nerve in rat

**DOI:** 10.1515/tnsci-2020-0112

**Published:** 2020-08-17

**Authors:** Fon-Yih Tsuang, Ming-Hong Chen, Feng-Huei Lin, Ming-Chia Yang, Chun-Jen Liao, Wen-Hsiang Chang, Jui-Sheng Sun

**Affiliations:** Institute of Biomedical Engineering, College of Engineering and College of Medicine, National Taiwan University, Taipei, Taiwan; Department of Surgery, National Taiwan University Hospital, Taipei City, Taiwan; Department of Surgery, Division of Neurosurgery, WanFang Hospital, Taipei, Taiwan; Institute of Biomedical Engineering, College of Engineering and College of Medicine, National Taiwan University, Taipei, Taiwan; Division of Medical Engineering, National Health Research Institute, Miaoli County, Taiwan; Orthopedic Device Technology Division, Industrial Technology Research Institute, Hsinchu County, Taiwan; Department of Orthopedic Surgery, College of Medicine, National Taiwan University, Taipei, Taiwan; Department of Orthopedic Surgery, National Taiwan University Hospital, Taipei, Taiwan

**Keywords:** partial enzyme digestion, Liberase, nerve regeneration, rat model

## Abstract

Peripheral nerve injury is a life-changing disability with significant socioeconomic consequences. In this rat model, we propose that partial enzyme digestion can facilitate the functional recovery of a crushed nerve. The sciatic nerves were harvested and *in vitro* cultured with the addition of Liberase to determine the appropriate enzyme amount in the hyaluronic acid (HA) membrane. Then, the sciatic nerve of adult male Sprague-Dawley rats was exposed, crushed, and then treated with partial enzyme digestion (either 0.001 or 0.002 unit/mm^2^ Liberase-HA membrane). The sciatic function index (SFI) for functional recovery of the sciatic nerve was evaluated. After 2 h of *in vitro* digestion, fascicles and axons were separated from each other, with the cells mobilized. Greater destruction of histology structures occurred in the high enzyme (Liberase-HA membrane at 0.002 unit/mm^2^) group at 24 h than in the low enzyme (0.001 unit/mm^2^) group at 48 h. In the SFI evaluation, the improvement in 0.001 unit/mm^2^ Liberase group was significantly better than control and 0.002 unit/mm^2^ Liberase group. Our study demonstrated that appropriate enzyme digestion had a significantly faster and earlier recovery.

## 1 Introduction

Peripheral nerve injury (PNI) is a life-changing disability with significant socioeconomic consequences. Although when compared with the central nervous system (CNS), the peripheral nerve has a greater ability to repair or regenerate after injury, it remains a clinical challenge to restore normal function [1,2,3]. The restoration of nerve function is mainly dependent on the re-growth of the nerve itself. Peripheral nerves contain unmyelinated (sensory and autonomic) axons as well as myelinated (motor and sensory). After axon injury, Schwann cells (SCs) form bands of Büngner, secrete neurotrophic growth factors, and support axonal regeneration and re-myelination [4]. In this process, SCs can facilitate nerve regeneration; while the excessive extracellular matrix (ECM) and growth of fibrotic tissue during nerve regeneration also impede their abilities of nerve regeneration.

In the peripheral nervous system (PNS), SCs are the glial cells that play numerous supporting functions and the myelination of axons. SCs are also critical cellular targets for enhancing nerve regeneration. After PNS injury, SCs facilitate regeneration by phagocytosing cellular debris and provide physical and biochemical cues to guide axon growth [5]. In the crush injury of nerve, a significant amount of SCs dies, and the extracellular matrix is de-arranged. The surviving SCs migrate to form a band of Büngner as the guide for axonal re-growth. In adult nerve tissue, the dense ECM at the wound site may impede cellular migration and local proliferation, while the repair after PNI is further exacerbated and limited by SCs’ intrinsic hypocellularity [6,7].

The peripheral nerve injuries are classified according to the extent and severity of the injury [8,9,10,11]. In the first-degree injury (neurapraxia), the nerve compression comes with or without demyelination and usually results in complete recovery. The second-degree injury (axonotmesis) occurs when the axon is disrupted, but all the supporting elements (endoneurium, perineurium, and epineurium) remain intact. For the third-degree injury, the endoneurium is disrupted, but the perineurium and epineurium remain intact. In the fourth-degree injury, all neural and supporting elements are transected, but only the epineurium remains intact. The fifth-degree injury (neurotmesis) is characterized by the complete transection of the nerve. In the crushed model of our study, the endoneurial tubules are still intact. But, in the second- to fourth-degree injuries, intrafascicular fibrosis and then a barrier of the active glial cell wall produced may hinder the axonal re-growth pathway of its denervated targets. In clinical practice, direct end-to-end sutures are reserved for nerve injuries with small gaps; if proximal and distal nerve stumps cannot be opposed without tension, nerve grafting or the use of different materials of nerve conduits is considered [12].

Our institute had demonstrated that the cells released from the partially digested cartilage fragment could enhance the cartilage regeneration in the mouse model [13]. For the spinal cord injury, chondroitin sulfate proteoglycan is the major component of glial scars that restrict axonal regeneration in the lesion site. Huang and his colleagues also demonstrate that chondroitinase, a bacterial enzyme that breaks down chondroitin sulfate proteoglycan, can promote axonal re-growth across the injured site [14,15]. Until now, the treatment option for crushed nerve injury is still limited and it remains a clinical challenge to restore normal function.

In clinical medicine, a minor crushed nerve injury does not require direct surgical repair. In this study, we use minor crushed nerve injury model and hypothesize that partial digestion of the minor crushed nerve could reprogram the wound site and eliminate the biophysical impediments that might hinder cellular migration and proliferation.

## 2 Materials and methods

**Figure 1:**
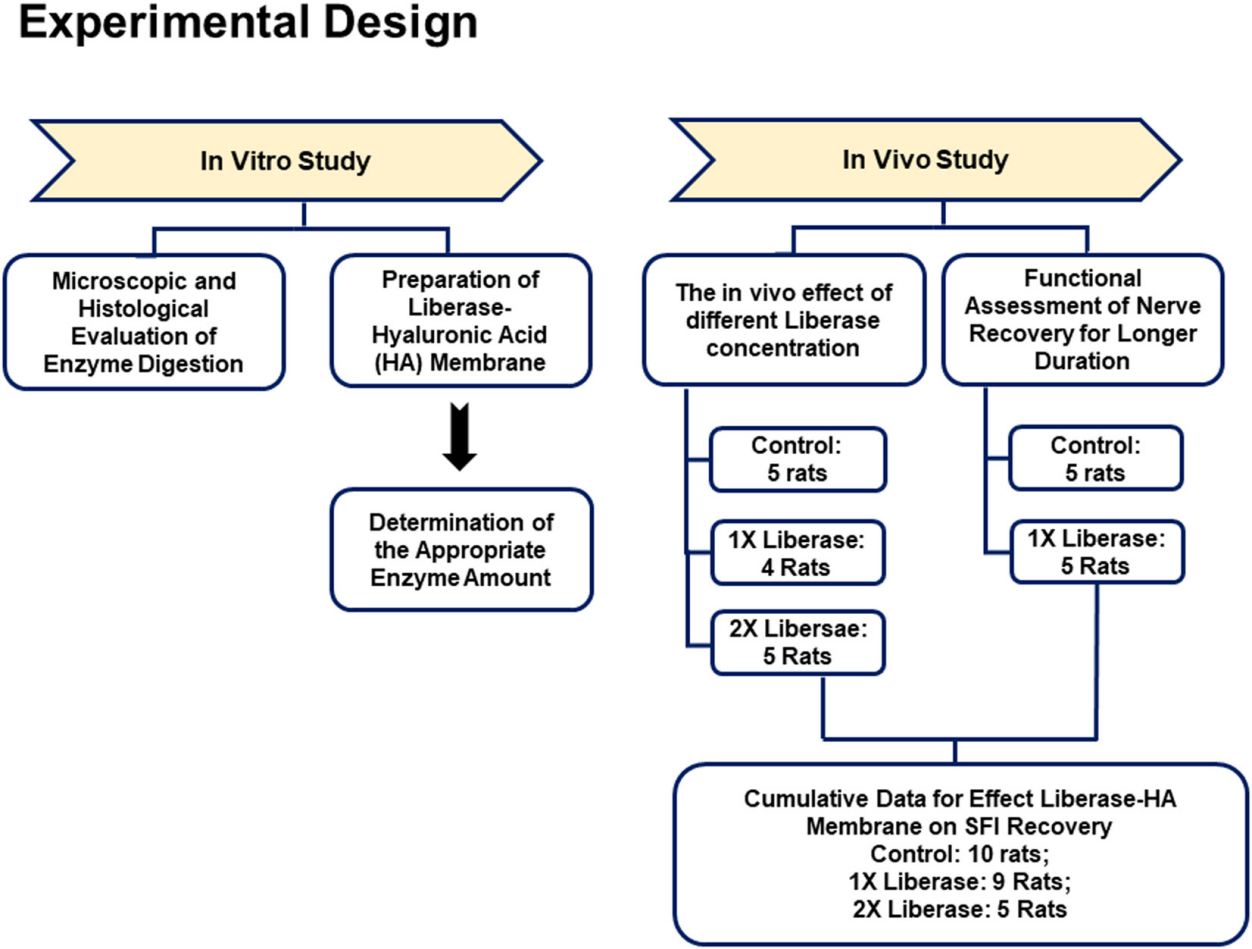
The flow-chart of experimental design.

This animal study was pre-approved by the Institutional Animal Care and Use Committee (IACUC) of Industrial Technology Research Institute (Hsinchu County, Taiwan) and National Taiwan University College of Medicine and College of Public Health (Taipei, Taiwan) (Affidavit of Approval of Animal Use: #ITRI-IACUC-2012-07). The experimental design of whole study is presented in Figure 1.

### 2.1 *In vitro* study

#### 2.1.1 Microscopic and histological evaluation of enzyme digestion

After euthanasia by overdose of ketamine hydrochloride injection, the sciatic nerves were harvested, stripped off the epineurium, and cultured in DMEM-F12 (Sigma-Aldrich Co.) with 10% fetal bovine serum (FBS; Thermo Fisher Scientific Inc., Vantaa, Finland) and 1% penicillin/streptomycin for 14 days. Then, the harvested nerve was cut into small pieces, approximately 0.3 mm in size, and cultured in regular medium with the addition of 0.74 unit/mL Liberase (Roche Diagnostics Corporation, Indianapolis, IN, USA). At 0 and 2 h after digestion, the sciatic nerve was fixed with 10% of formalin, embedded with paraffin section to 4-µm thickness, and stained with hematoxylin/eosin (H/E), and observed by light microscope for histological evaluation.

The morphological changes in the sciatic nerve grafts during the digestion process were observed at 0, 4, and 30 h after digestion. After 30-hour digestion, the specimens were rinsed with Hank’s balanced salt solution (HBSS; Thermo Fisher Scientific) to reduce enzyme activity, centrifuged at 235 rpm for 5 min, supernatants removed, and the specimens were then cultured in 6-well dishes. After 20 days’ culture, the dishes were rinsed with phosphate-buffered saline (PBS, Sigma-Aldrich Co.), and the cell clusters were then rinsed with 1 mL of iced DMEM to separate the SCs from the firmly attached fibroblasts. The DMEM (Sigma-Aldrich Co.) containing the SCs was centrifuged at 235 rpm for 5 min and moved to laminin- and poly-l-ornithine-coated dishes for 24 h; then DAPI and S-100 (Thermo Fisher Scientific Inc.) immunohistochemistry staining was applied and observed under an optical microscope (Axioplan**^®^**; Carl Zeiss, Germany). Quadruplicate samples were examined for each test. The number of mobilized SCs in the cell culture was counted and the influence of the different liberase concentrations (0.74 and 1.48 unit/mL Liberase) on the mobilized SCs was also compared. All enzyme solutions were prepared in combination with 50 μg/mL neutral red dye (Sigma-Aldrich Co.), a vitality dye that shows a deep red color in acid cell structures to express visualization of viable cells.

#### 2.1.2 Preparation of Liberase-hyaluronic acid (HA) membrane

For the 1X group, 26 units of Liberase (Roche Diagnostics Corporation, Indianapolis, IN, USA), 70 mL of deionized distilled water, and 1.575 g of hyaluronic acid (HA) (Industrial Technology Research Institute, Taiwan) were mixed at 4°C for 24 h (0.74 unit/mL), centrifuged at 1,200 rpm and 4°C for 10 min; then 2.5 mL of the mixed gel was laid flat onto a 3.5 cm-dish, sterile packed, and stored in a −80°C freezer for one night. After 10 days’ dehumidification in 4°C refrigerator, the Liberase-HA membrane was stored in a −20°C freezer for later use. The final Liberase concentration for the 1X group membrane was 0.001 unit/mm^2^ membrane. We also prepared a solution containing double the concentration of Liberase enzymes (1.48 unit/mL) for the 2X group (0.002 unit/mm^2^ membrane).

#### 2.1.3 Determination of the appropriate enzyme amount

After 24 h treatment with three different concentrations of Liberase-HA membrane (control, 1X: 0.001 unit/mm^2^, and 2X: 0.002 unit/mm^2^, in 5 × 5 mm membrane), the nerve segments of each group were observed at routine H/E staining and immunohistochemistry staining for type IV collagen. For the 1X group, another two nerve segments were observed at 48 h. Briefly, after 10% formalin fixation and paraffin embedding, the tissues were cut into sections of 4-µm thickness and stained with basic H/E stain and immunohistochemistry stain for type IV collagen. In the immunohistochemistry staining, the microscopic photographs of the type IV collagen staining were used for digital analysis. The area of interest (AOI) in the microscopic photographs was not only randomly but also evenly selected on the whole specimen and the green elements transformed into binary black/white elements through software. The black area represented stained type IV collagen, and the white area represented the background. The percentage of type IV collagen in the AOI was calculated.

### 2.2 *In vivo* study

#### 2.2.1 Design of animal experiment

Sixteen-to-twenty-week-old adult male Sprague-Dawley rats, weighing 250–400 g, were used. Rats were kept in individual cages with free access to specific rat chow and water prior to and after surgery. The cages were placed in a temperature- and humidity-controlled room with 12-hour light cycles. Animals were kept for 12 weeks postoperation to allow sufficient time for nerve regeneration and distal plantar muscle re-innervation. All procedures employed in this study were performed in accordance with the standards of guidelines for the care and use of laboratory animals established by the Industrial Technology Research Institute, Taiwan.

#### 2.2.2 Ethical agreement

This animal study was pre-approved by the Institutional Animal Care and Use Committee (IACUC) of Industrial Technology Research Institute (Hsinchu County, Taiwan) (Affidavit of Approval of Animal Use: #ITRI-IACUC-2012-07).

#### 2.2.3 Surgical procedure

The sciatic nerve crush injury model was performed, as described by a previous report [16]. Briefly, the mice were anesthetized by an intramuscular injection of a 1.1 mixture of ketamine (50 mg/mL; ketamine hydrochloride; Ketalar**^®^**, Pfizer, Taiwan) and xylazine (20 mg/mL; Rompun**^®^**, Bayer, Taiwan) at the dosage of 1 mL/kg (mixture/body weight). The lateral aspect of the right thigh was prepared and disinfected with 70% ethanol. A 1.5-cm posterolateral longitudinal straight skin incision was made to expose the right sciatic nerve. Then, the nerves were subjected to a calibrated sciatic crush injury at the middle of the right sciatic nerve, at approximately 10 mm from the trifurcation, by using a 5# jewelry forceps for 30 s. A crushed lesion of 1-mm length was produced. The crush site was marked with a single 8-0 Prolene**^®^** (Ethicon, Taiwan) epineural stitch at the distal limit of the injury for later identification [17]. The crushed sciatic nerve was put into the channel of a nerve conduit which was secured to epineurium with 8-0 Prolene**®** (Ethicon, Taiwan) stitches. The silicon nerve conduit, 3 mm in outer diameter and 1 mm in inner diameter, was pre-cut longitudinally. After the pre-designed membrane treatment was applied and wrapped around the crushed sciatic nerve, the nerve conduit was used to protect and keep the membrane in site. The nerve conduit was then closed, the soft tissue and skin was closed layer by layer. The animals in the control group underwent the same surgical procedure without membrane treatment applied inside the conduit. The entire surgical procedure was carried out under 5X magnification of a surgical loupe (EyeMag Pro F**^®^**; Carl Zeiss, Germany). In this study, we did not perform the sham surgery; the functional assessment of nerve recovery of all groups was normalized to the non-operated normal control rats.

#### 2.2.4 Determination of the appropriate enzyme amount

The purpose of the initial *in vivo* study was to determine the appropriate enzyme amount for effective Liberase digestion. In this part of study, 15 rats (5 rats in each group) in the non-treated control group and the study groups (1X and 2X Liberase concentrations) were designed. During the surgical procedure, either 1X or 2X Liberase-rinsed HA membrane was applied on the nerve injury site of the study groups. Recovery of the sciatic motor function was observed every week. The chosen concentration of Liberase-HA membrane (1X) was used to analyze functional assessment of nerve recovery for longer duration.

#### 2.2.5 Functional assessment of nerve recovery

The sciatic function index (SFI) was calculated using the widely accepted formula [18] to evaluate the functional recovery of sciatic nerve. To investigate the Liberase effect on sciatic nerve function recovery, 10 rats (5 rats each in non-treated control and 0.001 unit Liberase/mm^2^ membrane groups) were studied. The Liberase-HA membrane was used to cover the nerve injury site of the experimental group, as described above. By using walking tract analyses, the recovery of the sciatic motor function was observed and evaluated every week. Briefly, the animals were placed in a walking pathway ending in a darkened cage. All rats were first subjected to several conditioning trials. After they could walk steadily to the darkened cage, white paper was cut to the appropriate dimensions and placed at the bottom of the tract; the rat’s hind feet were dipped with Indian ink, and the animals were permitted to walk down the tract, leaving their hind footprints on the paper. Several measurements were taken from the footprint analysis: (1) the print length: distance from the heel to the third toe; (2) the toe spread: distance from the first to the fifth toe; and (3) the intermediate distance from the second to the fourth toe. Because self-mutilation was inflicted in some experimental animals, the measurements were performed at the distance between the most distal ends of the toes. All three measurements were taken for both the experimental (E) and normal (N: control) sides. The three factors contributing to the determination of the SFI were calculated as follows: (1) print length factor (PLF) = (EPL − NPL)/NPL; (2) toe spread factor (TSF) = (ETS − NTS)/NTS; (3) intermediate toe spread factor (ITF) = (EIT − NIT)/NIT. These factors were then incorporated into the Bain–Mackinnon–Hunter SFI formula [18,19,20]:

\text{SFI}=-38.3\times \text{PLF}+109.5\times \text{TSF}+13.3\times \text{ITF}-8.8

Regarding the index of recovery, an SFI of −100 indicates total sciatic nerve impairment, and a score of 0 represents a normal uninjured rat. SFI was used in this study as a quantitative assessment of hind feet function after sciatic nerve regeneration.

#### 2.2.6 Statistical analysis

All results were expressed as mean ± standard deviation of these experiments and statistically analyzed by two-way ANOVA. Statistical significance by Dunnett’s test was set at *p* < 0.05 between the means of the control and test groups. The unpaired two-tailed Student’s *t*-test was performed to compare differences between the groups for *in vitro* and *ex vivo* experiments. Differences were considered significant at *p* < 0.05. All analyses were performed using SPSS version 16.0 software.

#### 2.2.7 A statement of the location where the work was performed

All works were performed at the Institute of Biomedical Engineering, National Taiwan University, National Taiwan University Hospital (Taipei City, Taiwan), and Industrial Technology Research Institute (Hsinchu County, Taiwan).

## 3 Results

### 3.1 Histological evaluation of sciatic nerve grafts after digestion

**Figure 2:**
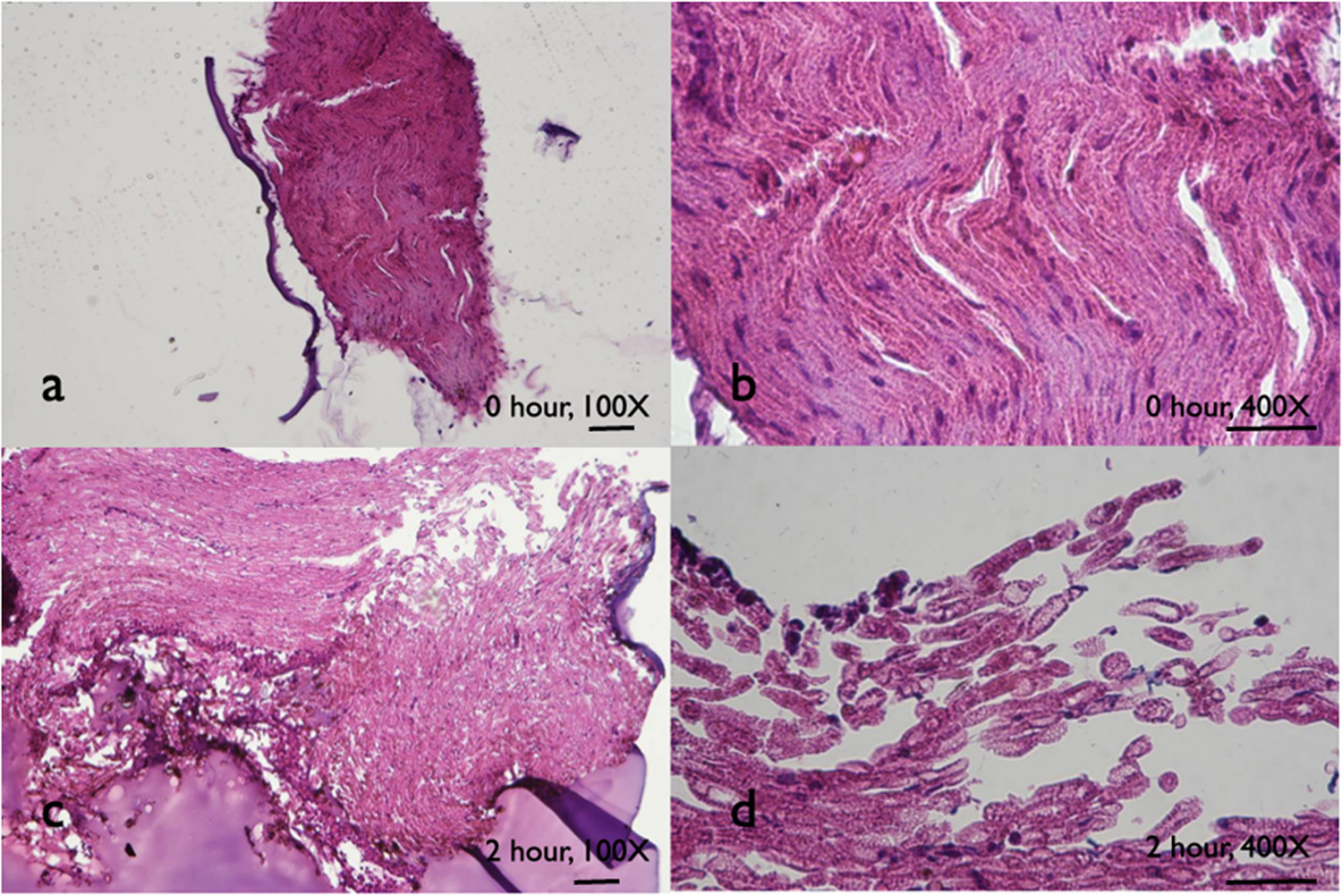
The histological evaluation of sciatic nerve grafts after digestion. H/E stain showed that, with 2 h of digestion, the nerve bundle structure became loose, and fascicles and axons were separated from each other (scale bar = 100 µm).

When nerve grafts underwent enzyme digestion for 0 and 2 h, axons were loosened, nerve bundle structure was disrupted, and the axons were separated from fascicles (Figure 2); when nerve grafts underwent longer enzyme digestion, the histological observation failed in the specimen because the specimens were too fragile to prepare for successful sections.

#### 3.1.1 SCs released from sciatic nerve grafts after digestion: *in vitro* evaluation

**Figure 3:**
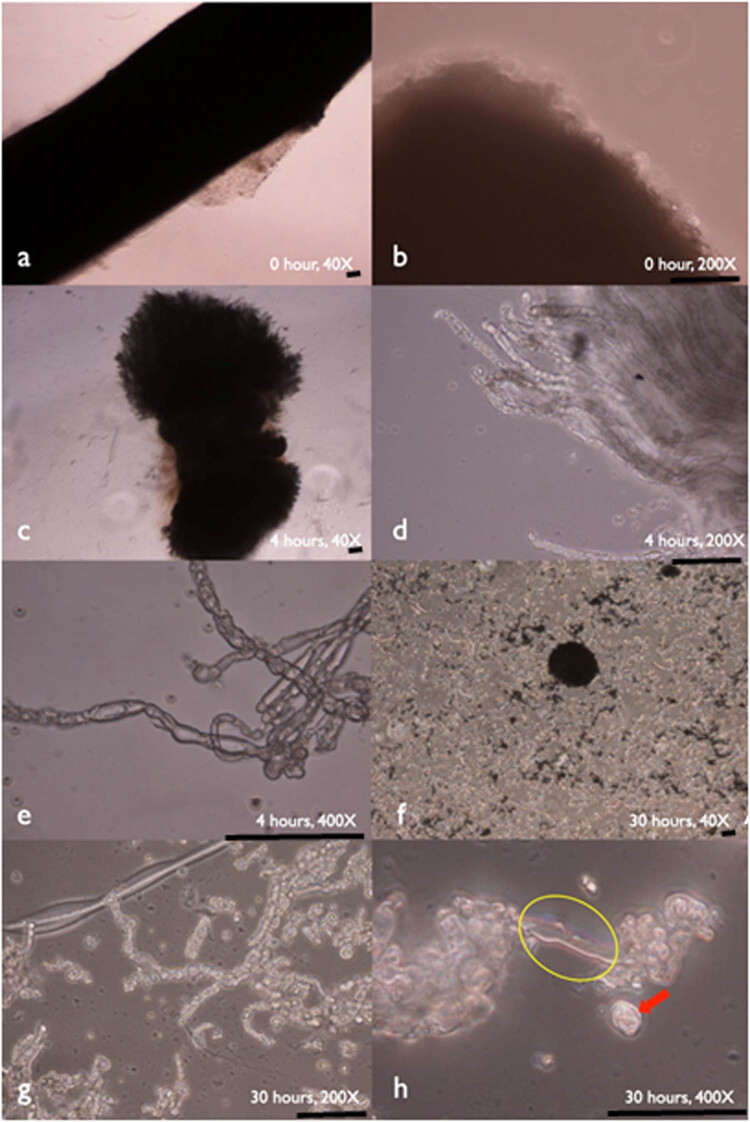
The microscopic morphology of the sciatic nerve grafts during the digestion process. At 0 h of enzyme digestion, the nerve endings were compact without individual axon visible (a and b). After 4 h after enzyme digestion, nerve ends became more loosened. Individual nerve axon can be observed (c and d); the axon with its attached SCs can be identified at higher magnification (e). After 30 h of digestion, cells are mobilized from their original histology structures. Exposed axon (yellow-circled) and cells mobilized from axon, possible SCs (red-arrowed), can be seen (f–h) (scale bar = 100 µm).

After digestion, the microscopic morphology of the sciatic nerve grafts during the digestion process was observed at 0, 4, and 30 h (Figure 3a–h). After 4 h enzyme digestion, the nerve ends became loose and axons with their attached cells (possible SCs) can be identified at a higher power view (Figure 3e). This indicated individual nerve axons released from nerve fascicles. After 30 h, the original nerve fascicle histology structures were disrupted. More cells were mobilized and migrated out from the extracellular matrix to adjacent region; and an exposed axon (yellow-circled), cells mobilized from axon (red-arrowed), and possible SCs can be seen (Figure 3h).

**Figure 4:**
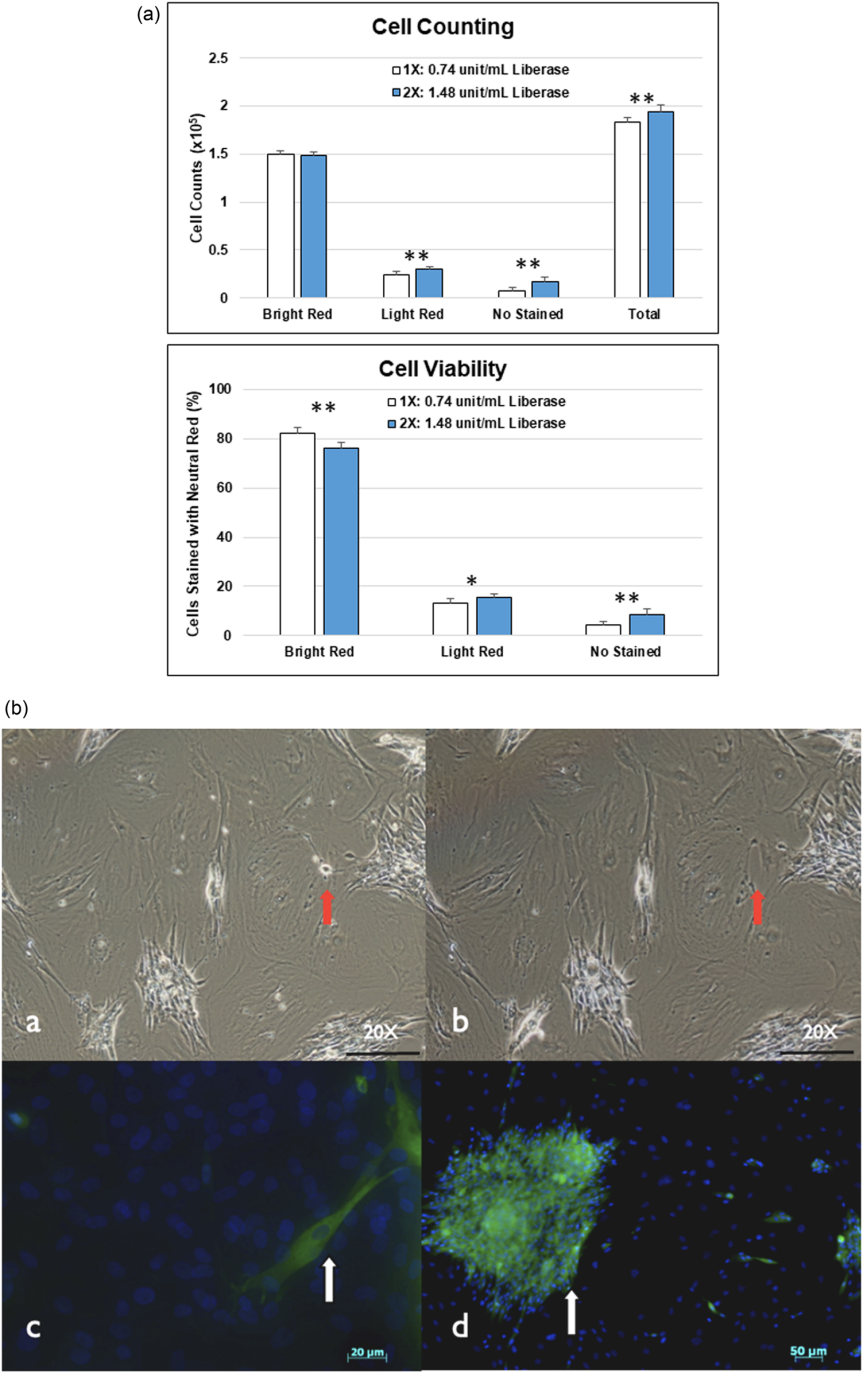
The quantitative and microscopic evaluation of cells released from sciatic nerve grafts after digestion. (a) Cell number and cell viability of mobilized SCs by different liberase concentration media. The total number of retrieved cells from different liberase concentrations differed significantly (*P* < 0.005). In comparison with the 1X liberase-treatment group (0.74 unit/mL Liberase: 1.83 ± 0.05 × 10^5^ cells), it was higher in the 2X liberase-treatment group (1.48 unit/mL Liberase: 1.94 ± 0.06 × 10^5^ cells). However, this difference in all treatment groups was not significant (*P* > 0.2) in the cell counts of viable cells according to their neutral red stain. Influence of the concentration of liberase-treatment group on the viability of cells was even more obvious when it presented as the ratio of cells stained with neutral red; the viability of 2X liberase-treatment group (76.3 ± 2.3%) was significantly lower than that of 2X liberase-treatment group (82.2 ± 2.1%) (*P* < 0.005). (*N* = 9; *: *P* < 0.05, **: *P* < 0.005) (*N* = 9; *: *P* < 0.05, **: *P* < 0.005). (b) The microscopic evaluation of cells released from sciatic nerve grafts after digestion. There were some smaller and densely packed cells visible before cold irrigation (a); after cold irrigation, those cells weakly adhered to dishes were collected (red-arrowed) (b). Positive cytoplasmic staining (green) of S-100 demonstrated the viable SCs; while other cells showed positive DAPI nuclear staining (blue) in the background (c and d) (scale bar in A & B = 100 µm).

The total number of retrieved cells from different liberase concentrations differed significantly (*P* < 0.005). In comparison with the 1X liberase-treatment group (0.74 unit/mL Liberase: 1.83 ± 0.05 × 10^5^ cells), it was higher in the 2X liberase-treatment group (1.48 unit/mL Liberase: 1.94 ± 0.06 × 10^5^ cells). However, this difference in all treatment groups was not significant (*P* > 0.2) in the cell counts of viable cells according to their neutral red stain (Figure 4a). The main contribution of total cell counts derived from the non-vital cells in the 2X liberase-treatment group. Influence of the concentration of liberase-treatment group on the viability of cells was even more obvious when they presented as the ratio of cells stained with neutral red; the viability of 2X liberase-treatment group (76.3 ± 2.3%) was significantly lower than that of 2X liberase-treatment group (82.2 ± 2.1%) (Figure 4a; *P* < 0.005).

We collected the released cells and cultured for 20 days in 6-well dishes. These cells proliferated and approached confluence. As fibroblasts adhered to the dishes prior to and stronger than the SCs, cold rinse was used to remove the SCs that were weakly adhered to the dishes (Figure 4b: a–b); we collected the loosely attached cells and cultured in the dishes pre-coated with laminin and poly-l-ornithine. After one day of culture, the cells were observed with DAPI and S-100 immunohistochemistry staining (Figure 4b: c–d). Positive S-100 staining demonstrated that viable SCs were successfully collected from enzyme-digested nerve tissue.

#### 3.1.2 Determination of the appropriate enzyme concentration

**Figure 5:**
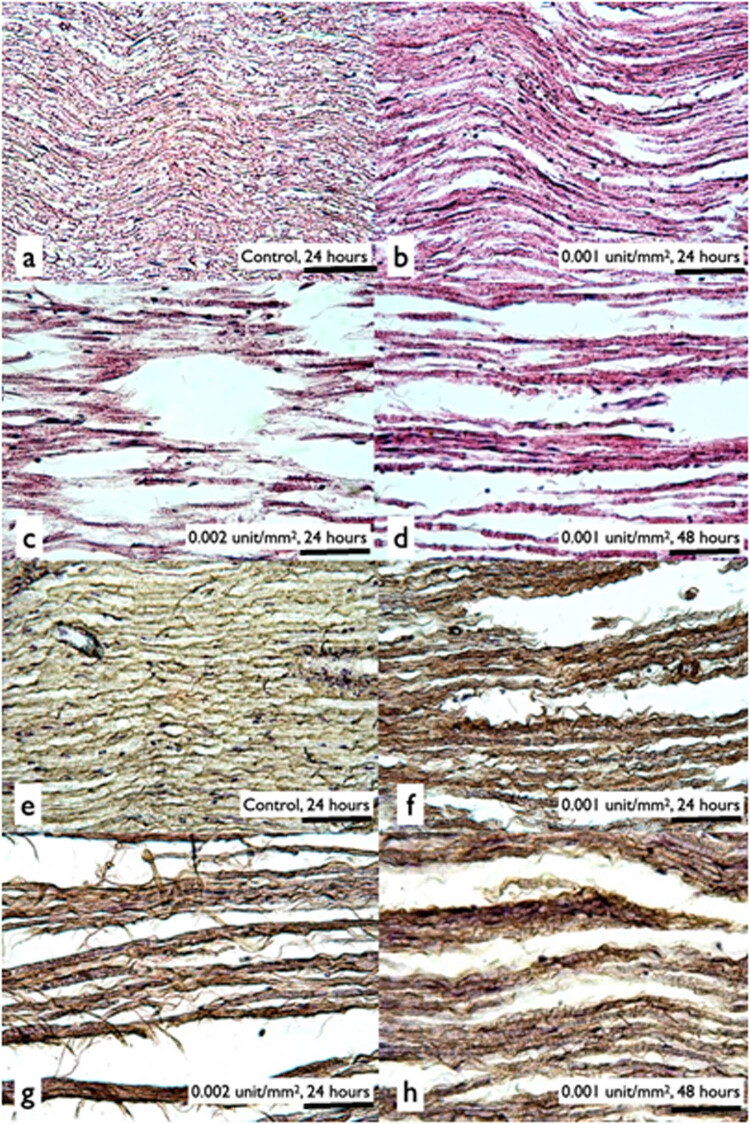
The effect of different Liberase concentrations. Liberase enzyme loosened the gross structure of the nerve graft by digested collagens. The results showed that more collagen was digested by the Liberase-HA membrane at 0.002 unit/mm^2^ for 24 h (c and g) than at 0.001 unit/mm^2^ for 48 h (d and h). (a–d) H/E stains; (e–h) type IV collagen stain. (b and f) Liberase-HA membrane at 0.001 unit/mm^2^, 24 -h digestion; (c and g) Liberase-HA membrane at 0.002 unit/mm^2^, 24 -h digestion; (d and h): Liberase-HA membrane at 0.001 unit/mm^2^, 48 -h digestion (200×) (scale bar = 200 µm).

In longitudinal sections of the control group, the nerve fibers displayed the standard parallel disposition to the long axis of the nerve, which was consistent with normal nerves with a parallel pattern of wavy fibers (Figure 5a and e); while the nerve segments that underwent enzyme treatment exhibited looser nerve bundle structures (Figure 5b–d). Immunohistochemistry staining analysis showed that nerve grafts subjected to greater concentrations of enzyme and longer enzyme treatment experienced more type IV collagen degradation than the control group (Figure 5f–h). In both H/E and immunohistochemistry staining, greater destruction of histology structures occurred in the 2X enzyme (0.002 unit/mm^2^ Liberase-HA membrane) group at 24 h than in the 1X enzyme (0.001 unit/mm^2^) group at 48 h.

**Figure 6:**
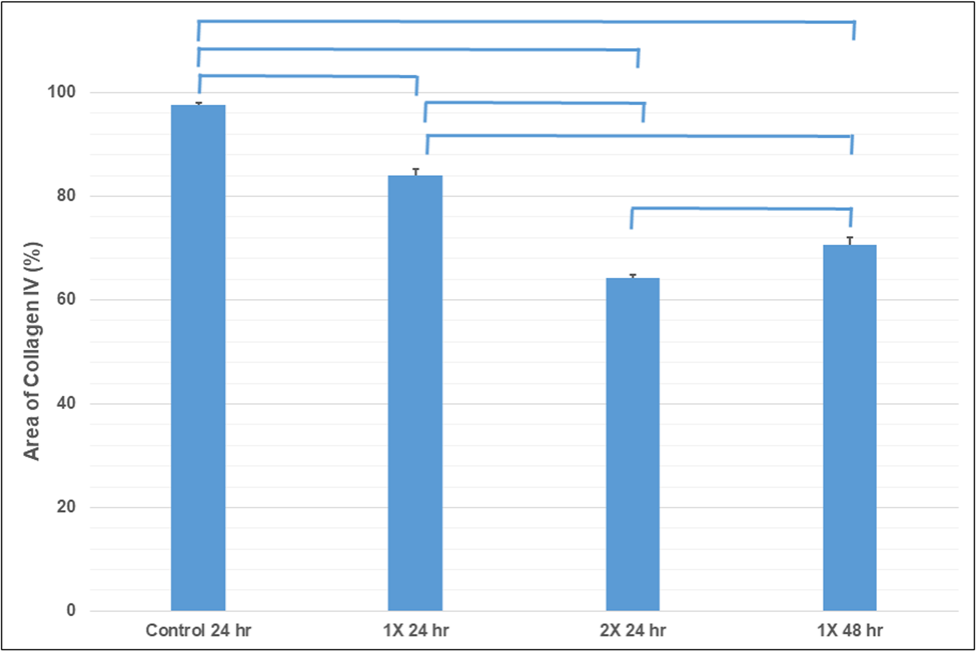
Digital analysis of the type IV collagen. After 24-hour digestion, type IV collagen amount of the 1X enzyme (Liberase-HA membrane at 0.001 unit/mm^2^) group was 13.9% less than that of the control group. While for the 2X enzyme (0.002 unit/mm^2^) group, it was 34.2% less than that of the control group. In addition, the 2X enzyme group digested statistically more collagen in 24 h than the 1X enzyme (0.001 unit/mm^2^) in 48 h.

The collagen amount was estimated quantitatively (Figure 6). After 24-hour digestion, type IV collagen amount of the 1X enzyme (Liberase-HA membrane at 0.001 unit/mm^2^) group was 13.9% less than that of the control group. While for the 2X enzyme (0.002 unit/mm^2^) group, it was 34.2% less than that of the control group. In addition, the 2X enzyme group digested statistically more collagen in 24 h than the 1X enzyme (0.001 unit/mm^2^) in 48 h.

#### 3.1.3 Liberase-HA membrane at appropriate concentration can facilitate SFI recovery

**Figure 7:**
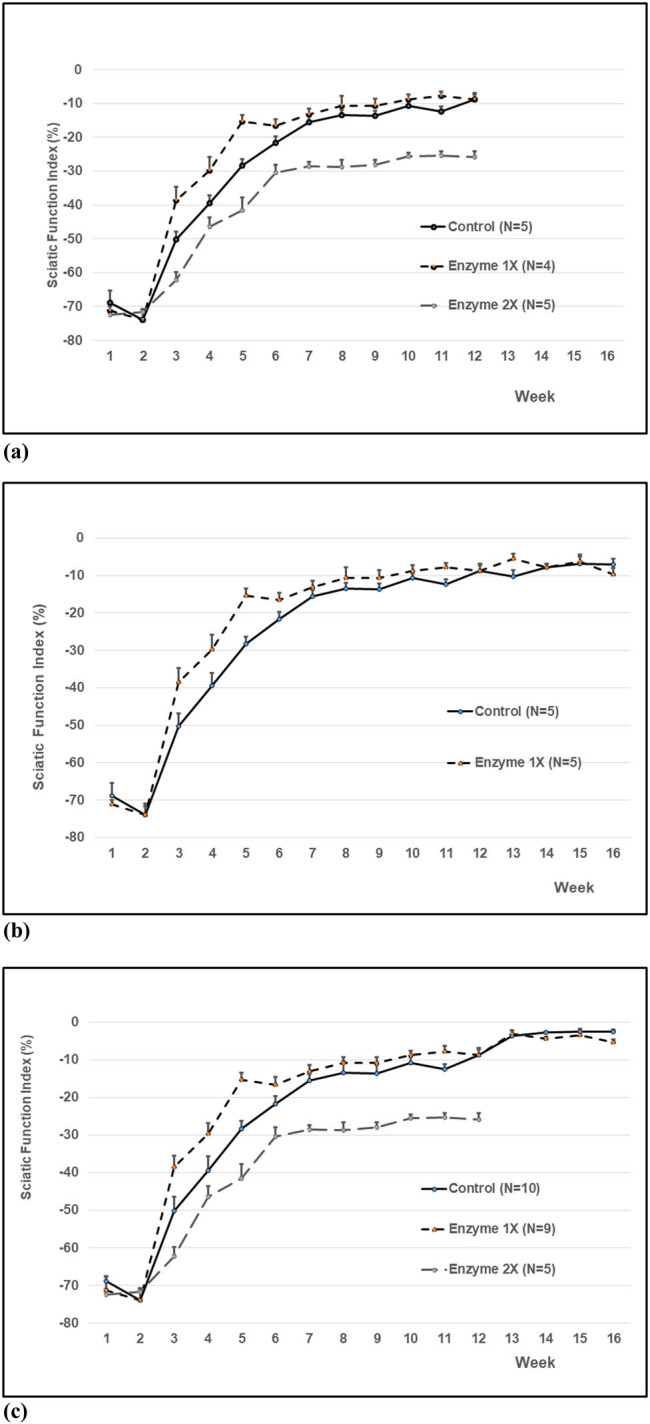
The *in vivo* effect of partial enzyme digestion on crushed nerve regeneration. (a) The *in vivo* effect of different Liberase concentrations. The improvement in enzyme 1X group (Liberase at 0.74 unit/mL) was significantly better at 3, 4, 5, 6, and 11 weeks after the injury (*p* < 0.05) than the control and enzyme 2X group (1.48 unit/mL). The functional results of the control group were significantly better than those of the enzyme 2X group throughout the whole test period. (b) Liberase-HA Membrane Facilitated SFI Recovery. There had been a significantly better improvement for enzyme digestion since 3 weeks after the injury. However, after 7th week, there was no recovery difference between control and enzyme digestion group, except at the 11th and 13th weeks. (c) Cumulative Data for Effective Liberase-HA Membrane on SFI Recovery. There had been a significantly better improvement for 1X enzyme digestion than that of control since 3 weeks after the injury; however, there was no recovery difference between control and enzyme digestion group after the 12th week. While the effect of 2X enzyme digestion seems inferior to that of control and 1X enzyme digestion group (control group: *n* = 10 before 12th week; *n* = 5 at 13th–16th week; 1X enzyme digestion group: *n* = 9 before 12th week; *n* = 5 at 13th–16th week; 2X enzyme digestion group: *n* = 5 before 12th week; *n* = 0 at 13th–16th week).

Initially, there were five rats in each group (non-treated control, 1X Liberase: 0.001 unit/mm^2^ membrane, and 2X Liberase: 0.002 unit/mm^2^ membrane). However, one rat in the enzyme 1X Liberase group had to receive euthanasia because of self-amputation of its toes at the experimental limb. Two weeks after surgery, a postoperative improvement for each rat of these three groups was observed. SFI versus time is plotted in the graph shown in Figure 7a. The improvement in the 1X Liberase group was significantly better at 3, 4, 5, 6, and 11 weeks after the injury (*p* < 0.05) than that in the control and 2X Liberase group. There was also significant difference that existed between the control and 2X Liberase group. The SFIs of all three groups reached plateaus after 8 weeks, which were −8 to −13% of pre-operative SFI for the control group; while they were −8 to −10% and −25 to −28% of pre-operative SFI for the 1X and 2X group, respectively. The injured nerve treated with the 1X Liberase group had a faster recovery course than the 2X Liberase group but not better neurological outcomes than that of control (Figure 7a).

The chosen concentration of Liberase-HA membrane (1X) was then analyzed for longer duration. There were five rats in each of the two groups: the non-treated control and 1X enzyme (Liberase-HA membrane at 0.001 unit/mm^2^) groups. SFI versus time is plotted in the graph shown in Figure 7b. Similar to the previous *in vivo* study (Figure 7a), neurological recovery had been observed postoperatively after 2 weeks, and the improvement in the enzyme group was statistically better (*p* < 0.05) after the third week. After 8 weeks, the improvement in both groups was slow, reaching a stationary plateau at −8.8% of SFI (Figure 7b).

The cumulative data for effective Liberase-HA membrane on SFI recovery showed that the 1X enzyme digestion group had a significantly better improvement than that of control since 3 weeks after the injury; while after the 12th week, there was no significant difference between the final neurological performances between the two groups. The effect of 2X enzyme digestion seems inferior to that of control and 1X enzyme digestion group (Figure 7c).

## 4 Discussion

PNI is a life-changing disability with significant socioeconomic consequences. Injury of a peripheral nerve leads to the degeneration of the distal nerve stump. During the first few days, SCs at the injured site create a microenvironment to allow nerve fiber re-growth from the proximal nerve ends by down-regulating the myelin genes, de-differentiation, proliferation, and then extrusion of their myelin sheaths [21,22,23,24,25,26,27]. SCs respond to the injured axon promptly by sequestering myelin debris and fragmenting their own myelin within two days [28,29]. Three days after the initial extrusion of myelin sheaths, SCs proliferate and line up within the basal lamina tube to form bands of Büngner, which later provide guidance cues for axonal regeneration [28]. Our study demonstrated that viable SCs could be mobilized from the nerve bundle structure (Figure 3). Early enzyme digestion may facilitate the release of SCs from the injured site to improve nerve regeneration, particularly during the first two days after the injury.

Peripheral nerve fibers regenerate from the proximal stump and that the speed of axonal re-growth was 3–4 mm/day after crushing a nerve, but only 2.5 mm/day after transection [30]. In this study, we also found that when freezing was used to eliminate SCs, the re-growth rate of a crushed nerve was the same, but the re-growth rate of a sectioned nerve was slowed to 1.2 mm/day. We proposed that depletion of SCs did not influence axonal elongation when the basal lamina remained in continuity, suggesting that the basal lamina tube at the lesion site may facilitate the action of neurotrophic factors from distal origin. As the basal lamina tube in our crushed nerve model was intact, we could propose that non-mobilized SCs help axonal regeneration to a limited degree; but the presence of mobilized SCs may significantly facilitate axonal regeneration.

Liberase has been the preferred collagenase blend used in human pancreatic islets’ isolation for more than ten years [31] and has been commercially available since the beginning of 1995. Liberase is purified from the culture supernatants of *Clostridium histiolyticum* grown in medium broth made from bovine brain and porcine heart, raising some concerns about infectious prions causing bovine spongiform encephalopathy in recent years. However, because this is an animal study, we decided to use this well-accepted enzyme to investigate the enzyme treatment hypothesis.

Although the PNS has more regenerative capacities than the CNS, patients with PNS disorders have far less satisfactory outcomes than data suggested from experimental animal models. For neurotmesis, treatment mainly consisted of either direct end-to-end surgical reconnection of the damaged nerve ends or the use of nerve grafts. In less severe cases, such as axonotmesis, where axon integrity is disrupted, but the continuity of the endoneurium sheath is preserved, patients receive mainly conservative treatments; and spontaneous regeneration through the distal nerve stump can restore normal nerve functions in this type of injury [9,10,11]. Even without any treatment, most of the recovery of neurological parameters reached a plateau at 6–8 weeks after the injury [32,33,34]; and an earlier rat study even showed normal walking track patterns returned to normal by 4 weeks postoperatively [35]. After nerve injury, the SCs convert themselves to a repair-promoting phenotype which allows them to adopt a strikingly adaptive response and to promote regeneration. They activate a sequence of supportive functions that engineer myelin clearance, prevent neuronal death, and help axon growth and guidance [36]. This may protect the denervated end organ, preserve the initial connection with proximal stump, and enhance the regenerating axons to grow from the proximal stump to the distal nerve stump [37].

Closing the nerve conduit might squeeze the rinsed HA sponge, cause loss of the enzyme contents, and we will remain uncertain about the actual amount of active enzyme retained inside the nerve conduit during the regeneration process. Therefore, we proposed another delivery form: the enzyme-containing HA membrane in silicon nerve conduit, which could gelatinize when in contact with body fluids. This form of enzyme treatment is more reliable for retaining the desired amount of enzyme inside a nerve conduit. In our *in vivo* study, we applied the enzyme treatment to the nerve injury site by a HA membrane with different enzyme contents. But another question might be raised, whether the enzyme might denature or lose its activity due to temperature changes during membrane preparation; the results of the *in vivo* studies were quite similar regarding the SFI recovery at similar enzyme concentrations, even with different enzyme treatment applications.

The results of our study also demonstrated that the SFI of either the non-treated control or enzyme digestion group reached a plateau at approximately 7–8 weeks postoperatively, and there was no significant difference between the two groups thereafter. However, we could observe that 1X enzyme digestion group had a significantly faster and earlier recovery course (Figure 7a–c). Although we could not observe any final functional difference between the non-treated control and 1X enzyme digestion groups in this rat model; but a faster and earlier recovery course in clinical practice for human patients may indicate a better opportunity for rehabilitation. An earlier return of muscle strength and anti-gravity exercise could be performed to prevent muscle atrophy and join contractures.

In our *in vivo* study, the SFI recovery of the 2X enzyme group was not only slower but also worse than that of the 1X enzyme group. In both the initial study (Figure 7a) and the cumulative data (Figure 7c), the results of the 2X enzyme group were even inferior to those of the non-treated control group. In the *in vitro* study, although the total number of retrieved cells from 2X liberase-treatment group was higher, its viability of cells was significantly lower than that of the 1X liberase-treatment group (Figure 4a). According to our *in vitro* digital analysis of the collagen amounts during the digestion process, the enzyme amount and digestion time were not linear to the digestion amount of collagen. The 2X enzyme group digested more type IV collagen in 24 h than the 1X enzyme group did in 48 h (Figure 5). We proposed that the results of initial study (Figure 7a) were given because the faster and stronger digestion effects in the early phase caused too much digestion of the histology structure, disrupting the tissue continuity. Once the tissue continuity is not strong enough to resist the tension at the nerve injury site, the loss in the tensile strength might interfere with nerve regeneration, even causing nerve tears at the injury site, which may deteriorate the beneficial effect of enzyme digestion on SFI recovery.

In the present work, the main purpose of this study is to prove concepts. We used two concentrations (1X and 2X) of the Liberase to determine the better enzyme amount in this experiment. Obviously, this may not be the most appropriate concentration; a future study is needed to perform a comparative evaluation of the enzymatic efficiency of Liberase and even effectiveness of other dissociation enzymes is also mandatory.

## 5 Conclusion

Through a series studies of *in vitro* and *in vivo* rat crushed nerve models, our findings show that partial enzyme digestion can loosen fascicle bundles, de-arrange extracellular matrix, facilitate Schwann cell mobilization, re-align Schwann cell to form the guidance for axon re-sprouting, and improve nerve repair by creating a more compliant microenvironment that expedites cell migration to and/or proliferation at the injury site. However, the utilization of enzyme digestion warrants further examination and research in future studies on nerve regeneration.
